# Editorial: Gliopathies in aging-related brain diseases: From understanding to therapy

**DOI:** 10.3389/fnins.2022.1017877

**Published:** 2022-09-14

**Authors:** Omar El Hiba, Tiziano Balzano, Arumugam R. Jayakumar

**Affiliations:** ^1^Faculty of Sciences, University Chouaib Doukkali, EL Jadida, Morocco; ^2^HM CINAC (Centro Integral de Neurociencias Abarca Campal), Hospital Universitario HM Puerta del Sur, HM Hospitales, Madrid, Spain; ^3^Department of Obstetrics, Gynecology and Reproductive Science, Leonard M. Miller School of Medicine, University of Miami School of Medicine, Miami, FL, United States

**Keywords:** neuron-astrocyte interaction, neurodegenerative disease, microglia, astrocyte, aging, oligodendrocyte, pericyte, BDNF

Neuron–glia interaction plays a pivotal role in the maintenance of neural homeostasis in the central nervous system (CNS). While glial cells, in general, provide strong structural support, they are also actively involved in maintaining complex brain homeostasis by the release of neurotransmitters, and trophic factors, as well as clearing neuronal debris.

Glial cells can be subdivided into four major groups: (1) astrocytes, (2) microglia (3) oligodendrocytes, and (4) their progenitors NG2-glia (Jäkel and Dimou, 2017). These cells are normally associated with supportive roles including contributions to energy metabolism, synaptic plasticity, synaptogenesis, neurogenesis, and ion homeostasis, as well as immune defense. The normal glial function is generally affected during major aging and neurodegenerative diseases, such as Alzheimer's disease (AD), Parkinson's disease (PD), Amyotrophic lateral sclerosis (ALS), Multiple sclerosis (MS), and Frontotemporal Lobar Degeneration (FTLD). Emerging evidence also indicates the potential involvement of these glial cells, in addition to neuronal cell involvement, in various rare neurological conditions (Draoui et al., 2019; Cassina et al., 2021; Ravi et al., 2021; Schirmer et al., 2021; Goodman and Bellen, 2022).

This Research Topic, therefore, provides original research and important review articles exploring multiple aspects of astrocytes, microglia, and oligodendrocytes, during aging and its associated neurodegenerative diseases. Researchers have presented their work and views on the potential mechanisms involved in the glial cell activation and dysfunction that are critical for a broad spectrum of aging-associated neurological conditions.

The involvement of astrocytes in the neuropathology of aging and age-related neurodegenerative diseases is likely a consequence of both the loss of normal homeostatic functions and the gain of toxic functions (Phatnani and Maniatis, 2015). Accordingly, Huang et al. submitted a review wherein the authors generalized the basic role of reactive astrocytes in neurodegeneration. They summarized the relationships between astrocytes and pathogenic or risk genes in several neurodegenerative diseases, including those associated with AD, ALS, FTLD, and PD.

Microglia are considered the first line of defense in the CNS, being these cells vigilant to pathological alterations occurring in the brain. In that context, Muzio et al. discussed the role of microglia in neurodegenerative disorders and brain aging and provided mechanisms involved in microglial activation, its regulation, and its contribution to the development of those neurodegenerative conditions.

Oligodendrocytes are the most abundant glial population, designated to ensheath the axons and provide them with metabolic and trophic support. In the aging brain, clinical and experimental studies showed an overall decline in the brain white matter volume accompanied by myelin degenerative phenotype suggesting an altered oligodendrocyte normal functions. In that line, Valori and Neumann reviewed the neuropathological and genetic evidence of oligodendrocyte impairment (TDP-43/FUS-proteinopathies). Taking into consideration that TDP-43 is involved in accelerating age-dependent neuronal degeneration (Tsuiji et al., 2017).

Finally, two last contributions were original research articles that focus on (1) Rett syndrome (RTT), a severe X-linked dominant neurodevelopmental disorder and (2) ischemic brain injury. The first one was a study conducted by Kim et al. exploring the role of brain-derived neurotrophic factor (BDNF) whose level changes and signaling pathways have been identified. The authors have investigated the neuroprotective role of (BDNF)-secreting mesenchymal stem cells (MSCs) from the human umbilical cord in a mice model of RTT. They show promising results *in vitro* and *in-vivo*, in which BDNF-MSCs increased the number of synapses/cells in the hippocampus, cortex, and striatum of the mice with RTT. The second investigation by Wang et al. shows how pericyte deficiency is involved in greater brain injury, BBB breakdown, and neuronal degeneration in mice post-stroke. The authors also highlight pericyte loss seen during normal aging and in neurodegenerative disorders and therapeutic strategies (e.g., AD and PD).

Overall, these series of articles within the present Research Topic have brought several interesting data allowing the understanding of glia-neuron interactions in a wide range of neurological conditions. We expect that this topic would expand our knowledge on the biological basis of glia-neuron interactions and give exciting insights into new therapeutic approaches to various neurological disorders by targeting glial cells.

## Author contributions

All authors listed have made a substantial, direct, and intellectual contribution to the work and approved it for publication.

## Funding

This work was funded by JUAN DE LA CIERVA-INCORPORACIÓN (IJC2020-043918-I), MCIN/AEI/10.13039/501100011033, and European Union Next Generation (EU/PRTR to TB).

## Conflict of interest

The authors declare that the research was conducted in the absence of any commercial or financial relationships that could be construed as a potential conflict of interest.

## Publisher's note

All claims expressed in this article are solely those of the authors and do not necessarily represent those of their affiliated organizations, or those of the publisher, the editors and the reviewers. Any product that may be evaluated in this article, or claim that may be made by its manufacturer, is not guaranteed or endorsed by the publisher.
